# Sputum microbiota and inflammation at stable state and during exacerbations in a cohort of chronic obstructive pulmonary disease (COPD) patients

**DOI:** 10.1371/journal.pone.0222449

**Published:** 2019-09-17

**Authors:** Solveig Tangedal, Rune Nielsen, Marianne Aanerud, Louise J. Persson, Harald G. Wiker, Per S. Bakke, Pieter S. Hiemstra, Tomas M. Eagan

**Affiliations:** 1 Dept. of Clinical Science, Faculty of Medicine, University of Bergen, Bergen, Norway; 2 Dept. of Thoracic Medicine, Haukeland University Hospital, Bergen, Norway; 3 Dept of Pulmonology, Leiden University Medical Center, Leiden, The Netherlands; 4 Dept. of Microbiology, Haukeland University Hospital, Bergen, Norway; Imperial College London, UNITED KINGDOM

## Abstract

**Background:**

Exacerbations of chronic obstructive pulmonary disease (COPD) are debilitating events and spur disease progression. Infectious causes are frequent; however, it is unknown to what extent exacerbations are caused by larger shifts in the airways’ microbiota. The aim of the current study was to analyse the changes in microbial composition between stable state and during exacerbations, and the corresponding immune response.

**Methods:**

The study sample included 36 COPD patients examined at stable state and exacerbation from the Bergen COPD Cohort and Exacerbations studies, and one patient who delivered sputum on 13 different occasions during the three-year study period. A physician examined the patients at all time points, and sputum induction was performed by stringent protocol. Only induced sputum samples were used in the current study, not spontaneously expectorated sputum. Sputum inflammatory markers (IL-6, IL-8, IL-18, IP-10, MIG, TNF-α) and antimicrobial peptides (AMPs, i.e. LL-37/hCAP-18, SLPI) were measured in supernatants, whereas target gene sequencing (16S rRNA) was performed on corresponding cell pellets. The microbiome bioinformatics platform QIIME2^TM^ and the statistics environment R were applied for bioinformatics analyses.

**Results:**

Levels of IP-10, MIG, TNF-α and AMPs were significantly different between the two disease states. Of 36 sample pairs, 24 had significant differences in the 12 most abundant genera between disease states. The diversity was significantly different in several individuals, but not when data was analysed on a group level. The one patient case study showed longitudinal dynamics in microbiota unrelated to disease state.

**Conclusion:**

Changes in the sputum microbiota with changing COPD disease states are common, and are accompanied by changes in inflammatory markers. However, the changes are highly individual and heterogeneous events.

## Introduction

A myriad of bacteria and other microorganisms, collectively called the human microbiota, inhabits the human body. With modern marker-gene DNA-sequencing technology more knowledge of how bacteria affect the human host is rapidly being acquired. It was long believed that the lower airways were sterile, but recent studies have shown a present microbiota also in healthy subjects [1–3].

Chronic obstructive pulmonary disease (COPD) is characterized by chronic inflammation in the airways [4], and an increase in systemic inflammation [5, 6]. The cause of the inflammation has been unknown, but toxic effects of inhaled tobacco or other substances [7] and auto-immunity has been suggested [8].

A dramatic manifestation of COPD, the acute exacerbations [9] with potentially life-threatening airways obstruction, is most often seen in combination with symptoms of infection. Indeed, bacteria and viruses are believed to trigger most exacerbations [10, 11]. Traditionally this has been seen as single-agent infections, and one debate has been whether any such agent was acquired by contagion or an upswing of pre-existing colonizing agents [12]. Although most exacerbations are likely due to infections, it is suspected that environmental factors like air-borne pollution and air-temperature can trigger these episodes [10]. Thus, single-agent infections are unlikely explanations for all or the entire COPD exacerbation event.

We suggest that the chronic inflammation of COPD reflects a chronically distorted microbiota. And, that the COPD exacerbations may reflect an acutely imbalanced respiratory ecosystem, with an accompanying inflammatory response to this imbalance.

However, little information exists to date on the dynamics of the airways microbiota in COPD patients shifting from a steady state to a COPD exacerbation [13]. In the current study we examined the microbiota in 36 COPD patients from whom we had induced sputum samples collected both during the stable state and during COPD exacerbations. And in one particular patient prone to experience frequent exacerbations we assessed the temporal changes of the sputum microbiota over 36 months in six samples from stable state visits, and seven collected during exacerbations.

## Methods

### Study population

**Fig 1:**
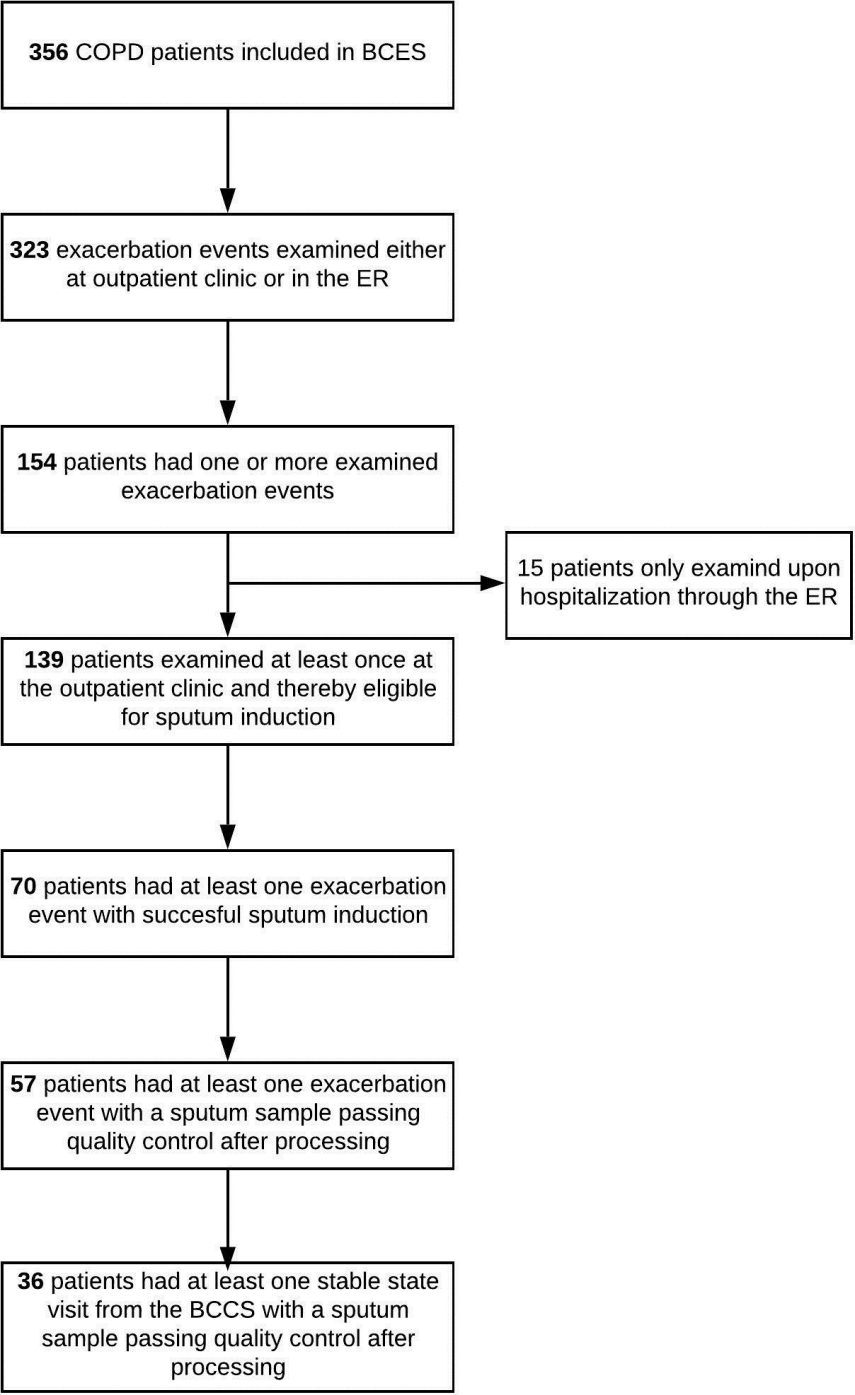
Flowchart depicting selection of the 36 patients in the current study, from the Bergen COPD Exacerbation Study (BCES) and the Bergen COPD Cohort Study (BCCS).

The Bergen COPD Exacerbation Study (BCES) included all COPD patients from the Bergen COPD Cohort Study (BCCS) that belonged to the Haukeland University Hospital district for emergency care (356 out of 433 COPD patients in the BCCS). Detailed descriptions of study design and inclusion for the BCCS and the BCES has been published [6, 14]. Only induced sputum samples were used in the current study, not spontaneously expectorated sputum. A flowchart depicting the selection of the study sample is presented in Fig 1. Of the 356 included patients, 154 had one or more examined exacerbation events. Sputum induction was attempted unless the patient declined or in some instances when we did not have available technicians to process the sputum fresh after the induction. A total of 36 patients had induced sputum of acceptable quality available both from a stable state and an exacerbation visit, and these 36 sputum pairs define the current study population (Fig 1).

All patients provided written informed consent, and the Norwegian Regional Ethical Committee approved the study (REK-Vest, case number 165.08).

### Data collection

A trained study physician examined all patients both at regular BCCS-visits and during BCES-exacerbation visits. Classification of airways obstruction was according to Global initiative for chronic obstructive lung disease (GOLD) guidelines [15]. Body composition was determined with bioelectrical impedance measurements, and patients categorized as normal, obese or cachectic [16]. COPD exacerbation history was taken by the study physician at the baseline visit of the study, based on patient recall. An exacerbation was defined as a worsening of symptoms requiring treatment with either antibiotics or oral steroids. Induced sputum sampling was performed depending on patients' cooperation and availability of study technicians trained in sputum processing.

### Laboratory analyses

Sputum samples had to fulfill quality measures ensuring lower airway sampling. The details of sputum induction and processing are previously published [14, 17].

Sputum processing was performed immediately after sampling. After the filtering step, samples were centrifuged at 4°C for > 15 minutes at 450 g. The resulting supernatants and cell pellets were frozen separately at -80°C. DNA was extracted from cell pellets using the FastPrep-24 Instrument and reagents from the FastDNA Spin Kit (MP Biomedicals, LLC, Solon, OH, USA). Amplicon PCR (45 cycles) and index PCR were run using primers from the Nextera XT Index Kit (Illumina Inc., San Diego, CA, USA). Paired-end sequencing (2 x 300 cycles) of the V3-V4 region of the 16S rRNA gene followed the protocol for Metagenomic Sequencing Library Preparation for the Illumina Miseq System (Part # 15044223 Rev. B, MiSeq Reagent Kit v3).

The inflammatory markers interleukin-6 (IL-6), interleukin-8 (IL-8), interleukin-18 (IL-18), interferon gamma-inducible protein-10 (IP-10), tumor necrosis factor-alpha (TNF-α) and monokine induced by gamma interferon (MIG) in sputum supernatants were processed using bead-based multiplex assays and the Luminex® xMAP® technology (Luminex Corporation, Austin, Texas). The data on sputum levels of LL-37 (a cathelicidin peptide derived from human hCAP-18) and secretory leucocyte protease inhibitor (SLPI) derived from previously unfrozen, aliquots of the same sputum supernatants by enzyme immunoassays, were derived from a previous analysis [18, 19].

### Bioinformatics analyses

The amplicon sequences were quality and chimera filtered through the microbiota pipeline Quantitative Insights Into Microbial Ecology 2 (QIIME2) (v.2017.9 – v.2018.11) [20], using the Divisive Amplicon Denoising Algorithm 2 (DADA2) [21]. Laboratory-made sequences (chimeras) were removed first through DADA2 [21] and then VSEARCH [22]. Negative controls were unavailable, so to filter contaminants we used the total DNA-load measurements (Quant-iT™ PicoGreen™, ThermoFisher Scientific Inc) and the Decontam algorithm in R [23]. Amplicon sequence variants (ASVs) created by DADA2 were assigned taxonomy, using a self-trained Naïve Bayes classifier and the Silva database [24]. ASVs that could not be assigned taxonomy beyond kingdom level were omitted. After de-novo alignment, FastTree was used to build a phylogenetic tree for diversity analyses [25].

### Statistical analyses

To compare inflammatory markers and antimicrobial peptides in sputum during the stable state and during exacerbations, Wilcoxon signed rank test was used to account for the paired design. To compare taxonomic composition between pairs of samples we calculated the Yue-Clayton measure of dissimilarity (1-θ_YC_) [26]. This was performed at the genus level, after omitting ASVs containing < 1% of the total amount of sequences. Differential abundances of taxa between disease states were analysed using an ANOVA-like differential expression procedure (Aldex2) in R [27]. Diversity analyses were performed after sub-setting all samples at the number of sequences of the sparsest sample (rarefaction). Beta-diversity visualized as non-metric multidimensional scaling plots (NMDS), were analysed with permutation tests of multivariate homogeneity of variances, permuted analysis of variance (PERMANOVA) and Procrustes analyses in the *Vegan* package in R [28]. For analyses of clinical data relative to measurements from biological samples StataSE (StataCorp LP. Release 14. College Station, TX) was used. Further details on bioinformatics and statistical methods are available in the online supplement S1 Text. More on bioinformatics and statistical methods.

## Results

Table 1 shows the patient characteristics for the 36 included COPD patients.

**Table 1:** Patient characteristics at inclusion in the Bergen COPD Cohort Study.

	n (%)
Sex	
Women	15 (42%)
Men	21 (58%)
Age	
40–54 years	4 (11%)
55–64 years	21 (58%)
65–75 years	11 (31%)
Body composition	
Normal	27 (75%)
Obese	6 (17%)
Cachectic	3 (8%)
Smoking	
Ex	21 (58%)
Current	15 (42%)
GOLD COPD stage	
II (FEV1 50–80%)	18 (50%)
III (FEV1 30–50%)	14 (39%)
IV (FEV1 <30%)	4 (11%)
Frequent exacerbator*	
No	24 (67%)
Yes	11 (31%)

* >1 exacerbation last 12 months prior to inclusion. One patient missing information. GOLD: Global Initiative for Chronic Obstructive Lunge Disease. COPD: Chronic obstructive lunge disease. FEV1: Forced expiratory volume 1st second

Eleven patients had experienced two or more exacerbations the last 12 months before inclusion. At inclusion 28 participants used inhaled corticosteroids. No patients used antibiotics or oral corticosteroids at stable state, whereas at exacerbation visits, one patient used antibiotics, one used oral corticosteroids, and one patient used both (Table 1). For 26 of the 36 sputum pairs, the stable sputum was collected prior to an exacerbation event, and vice versa for the other 10 pairs. The median number of days between the two collections were 257 days.

### Inflammatory markers and antimicrobial peptides

**Fig 2:**
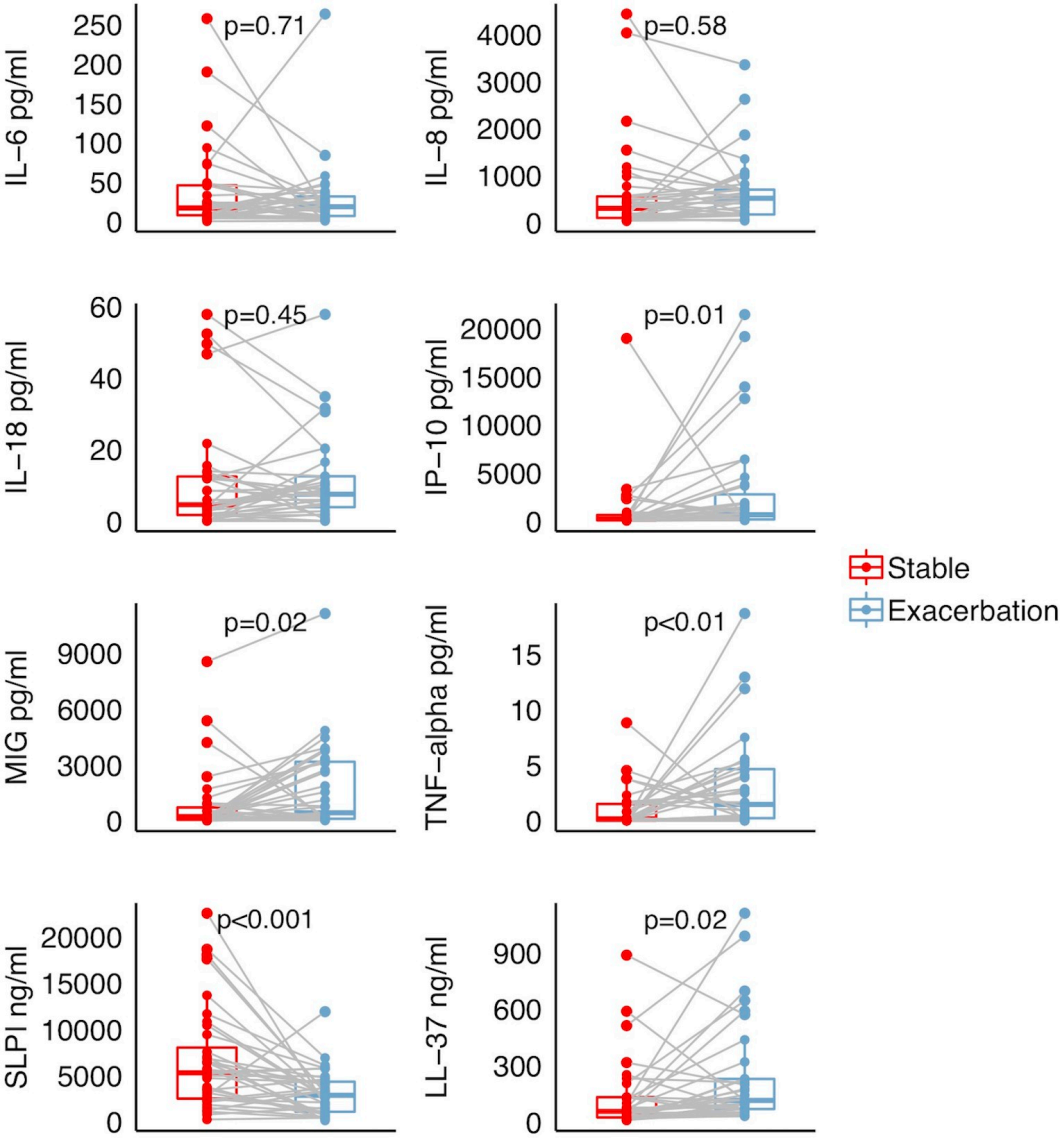
Inflammatory markers and antimicrobial peptides in induced sputum collected from a COPD cohort at stable state and during exacerbation. Interleukin-6 (IL-6), Interleukin-8 (IL-8), Interleukin-18 (IL-18), Interferon Gamma-Induced Protein 10 (IP-10), Monokine induced by gamma interferon (MIG): n = 35. Secretory Leukocyte Protease Inhibitor (SLPI): n = 36. LL-37/hCAP-18: n = 34. Boxes show the interquartile range (IQR = 75^th^ percentile-25^th^ percentile), with medians marked by the horizontal line within each box. Samples collected from the same patient at different disease states are connected by lines. Wilcoxon signed rank test was applied based on the paired, non-parametric data.

Levels of the two AMPs and three of the measured inflammatory markers (IP-10, MIG, TNF-α) differed significantly in sputum sampled between disease states (Fig 2), with levels of all mediators being higher during exacerbation except for SLPI. One patient had no measurement of inflammatory markers and two patients had no measurement of LL-37/hCAP.

### Taxonomy

**Fig 3:**
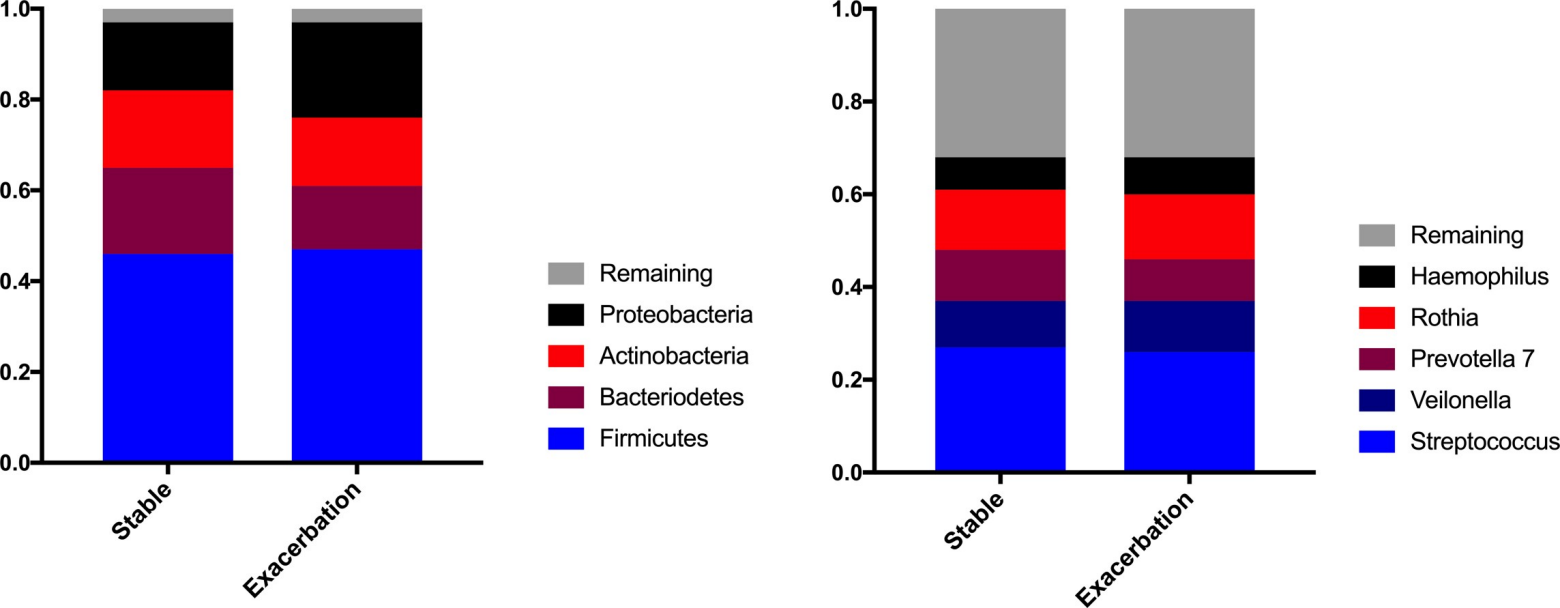
The four most abundant phylae and the five most abundant genera found in induced sputum samples from COPD patients during the stable state, and during exacerbations.

Of 15 phyla identified, *Firmicutes*, *Proteobacteria*, *Bacteroidetes* and *Actinobacteria* were the most abundant, containing 97% of all sequences at both disease states. *Proteobacteria* was relatively more dominating in samples collected during exacerbations compared to stable state. *Streptococcus*, *Rothia*, *Prevotella 7*, *Veillonella*, and *Haemophilus*; which altogether contained 68% of all sequences at both disease states were the most abundant genera (Fig 3).

The differential abundances of different taxa (often designated “features” in bioinformatics analyses) between disease states were tested at Silva’s phyla and genus level, and for each ASV. Differential abundances in features between the stable state-sample group and the exacerbation-sample group were not found (FDR-corrected, effect size cut off 0.5. Wilcoxon p>0.05 for all taxa at all three levels, all data available in S1 Table.).

**Fig 4:**
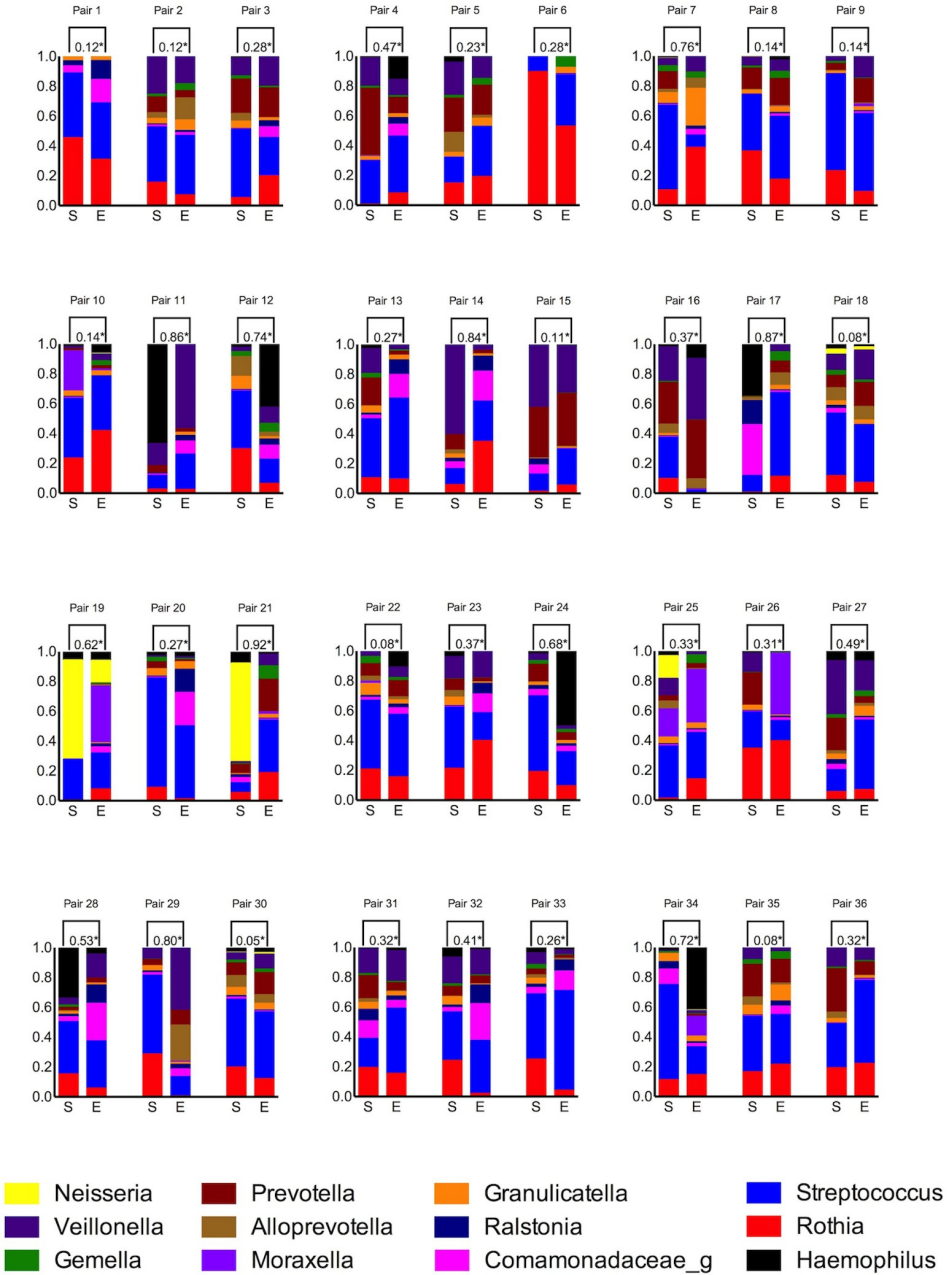
Comparison of bacterial composition in pairs of induced sputum samples (stable state and exacerbation) from 36 patients suffering from chronic obstructive lung disease. Presenting level 6 taxonomy (genus) provided by Silva database for amplicon sequence variants containing at least 1% of all sequences. *Yue-Clayton dissimilarity (1-θ_YC_) Range 0 to 1; 0 = perfect similarity, 1 = perfect dissimilarity. S: Stable state E: Exacerbation.

The taxonomic composition and 1-θ_YC_ of the 36 sputum pairs are shown in Fig 4. The Yue-Clayton measure is 0 with perfect similarity and 1 with perfect dissimilarity. To evaluate the similarity within each sputum pair, 0.2 was set as the Yue-Clayton limit for acceptable within-pair similarity. With this cut-off, 26 patients had sputum pairs considered dissimilar.

**Fig 5:**
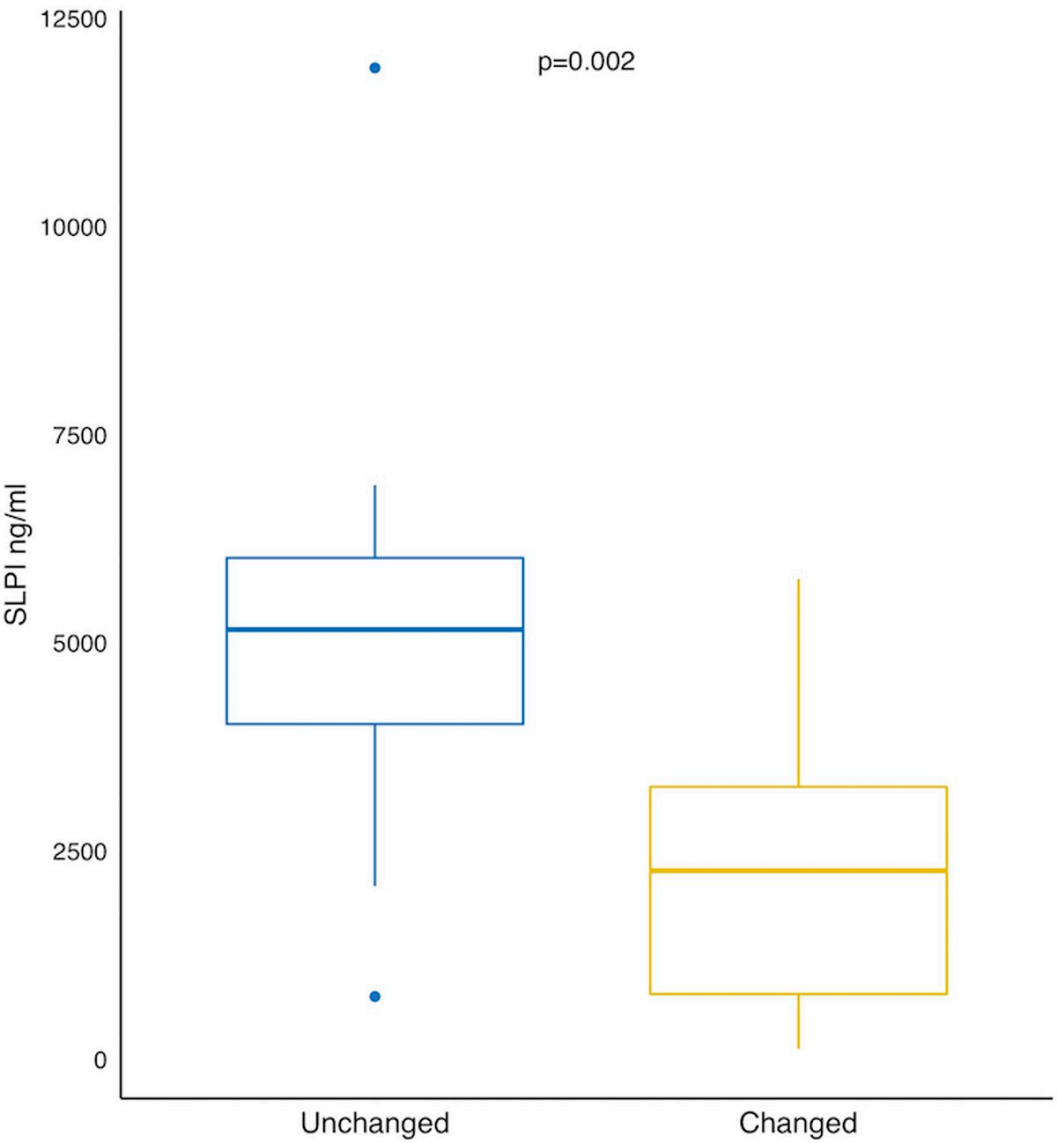
Comparing Secretory Leukocyte Protease Inhibitor (SLPI) measured in induced sputum in exacerbated COPD patients with regards to microbial composition alterations between disease states. Unaltered = Yue-Clayton dissimilarity index <0.2 (n = 10), Altered = Yue-Clayton dissimilarity index >0.2 (n = 26). Boxes show the interquartile range (IQR = 75^th^ percentile-25^th^ percentile), with medians marked by the horizontal line within each box. Kruskal Wallis test used due to non-parametric data.

The ten patients with low 1-θYC, and thus similar taxonomic composition across disease states, did not differ significantly from the other participants with regards to sex, age, body composition, smoking status, COPD stage, exacerbation frequency or use of inhaled corticosteroids (p>0.05, results not shown). Considering levels of inflammatory markers and AMPs at both disease states, only levels of SLPI during exacerbations were significantly lower in patients with dissimilar sputum pairs (Fig 5), whereas IL-8 trended towards higher levels in patients with dissimilar sputum during exacerbations (1-θ_YC_<0.2: Median IL-8 200.5 pg/ml, IQR (59.4–659.1) 1-θ_YC_≥0.2: Median IL-8 614.0 pg/ml, IQR (199.9–812.0), Kruskal Wallis p = 0.053).

### Diversity

Rarefaction curves of alpha-diversity (within-sample diversity) showed asymptote at 1000 sequences/sample (Fig 1 in S1 Text. More on bioinformatics and statistical methods). Faith’s phylogenetic diversity (PD) and Shannon’s non-phylogenetic diversity (non-PD) indices showed no significant differences in alpha-diversity between the two disease states (Table 1 in S1 Text. More on bioinformatics and statistical methods).

**Fig 6:**
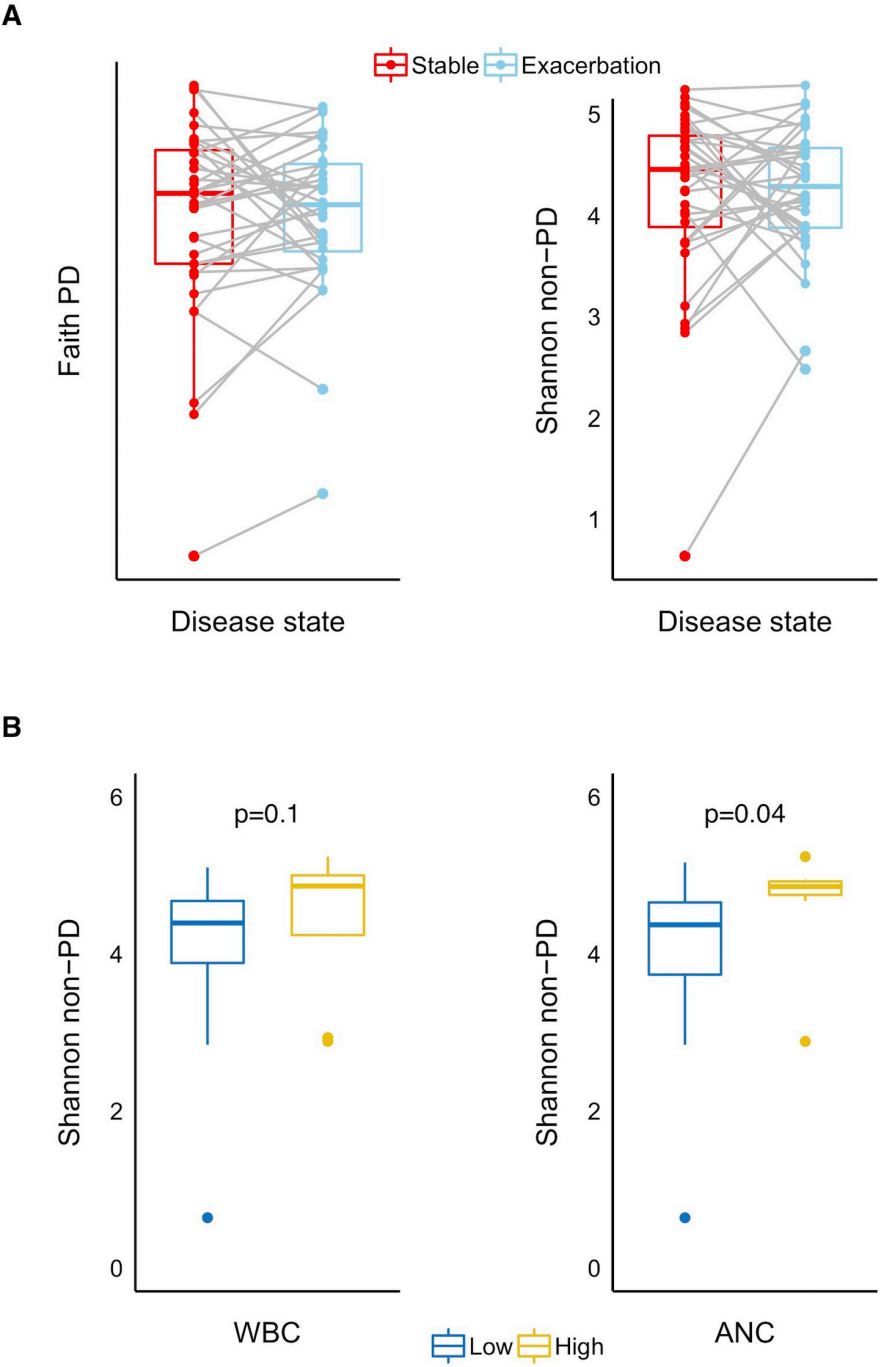
Alpha-diversity in induced sputum collected from 36 COPD patients. A: Comparison of phylogenetic (Faith PD) and non-phylogenetic (Shannon non-PD) alpha-diversity in sputum collected at stable state and during exacerbation. Lines connect samples from the same individual. Wilcoxon signed rank test was applied based on the paired, non-parametric data. B: Relations between Shannon’s alpha-diversity at stable state and serum inflammatory markers during exacerbations. WBC: White Blood Cell counts high >11.3 10^9^/L (n = 8) ANC: Absolute Neutrophil Count high >8.4 10^9^/L (n = 7). Kruskal Wallis test used due to non-parametric data. Boxes show the interquartile range (IQR = 75^th^ percentile-25^th^ percentile), with medians marked by the horizontal line within each box.

Changes in individual alpha-diversity between disease states are visualized in Fig 6. There were inconsistent directionality and magnitude of alpha-diversity change between patients. Faith’s PD was higher at stable state in 15 patients and for Shannon’s non-PD this was the case in 17 patients (Fig 6A).

Changes in Faith’s PD by disease state were not related to levels of white blood cell counts (WBC) or absolute neutrophil counts (ANC), while Shannon’s non-PD was lower at stable state among patients whose ANC did not become elevated during exacerbations (Kruskal Wallis, p = 0.04) (Fig 6B).

**Fig 7:**
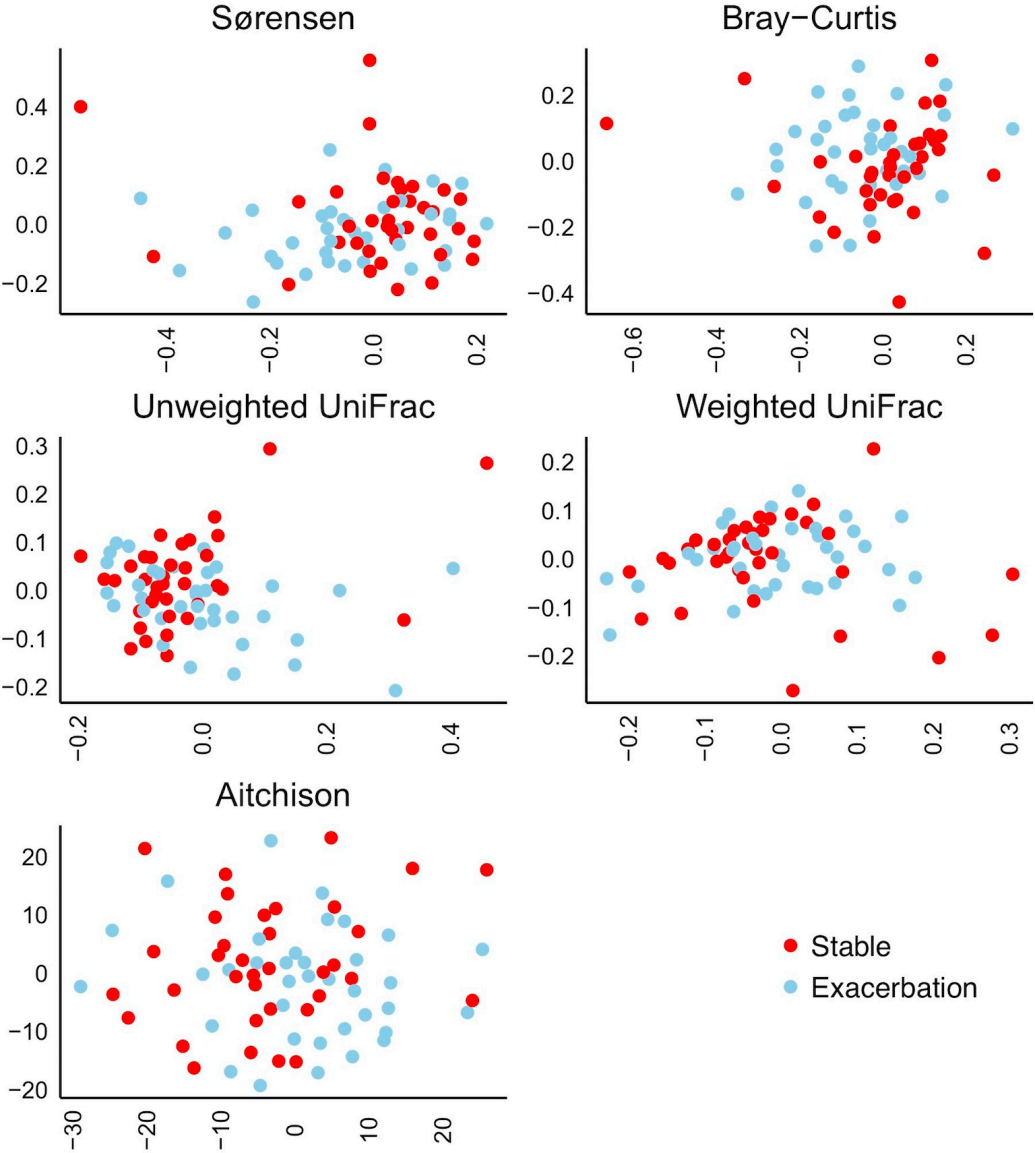
Beta-diversity in induced sputum collected from 36 chronic obstructive pulmonary disease sufferers both at stable state and during exacerbations, presented with non-metric multidimensional scaling (NMDS) ordinations. The X- and Y-axes display the first and second NMDS dimension respectively. Distance matrices: Sørensen and Bray Curtis: Both non-phylogenetic; qualitative and quantitative information respectively. Unweighted and weighted UniFrac: Both phylogenetic; qualitative and quantitative information respectively. Aitchison: Compositional interpretation of sequence counts.

We did not find clustering by disease state when we examined different ordinance plots of beta-diversity (between-sample diversity) (Fig 7). With the PERMANOVA test to compare the average community value (centroid) between disease-states, significant differences were found only for non-phylogenetic matrices (Bray-Curtis p = 0.017, and Sørensen p = 0.004), however the corresponding R^2 values were only 0.02 for both.

**Fig 8:**
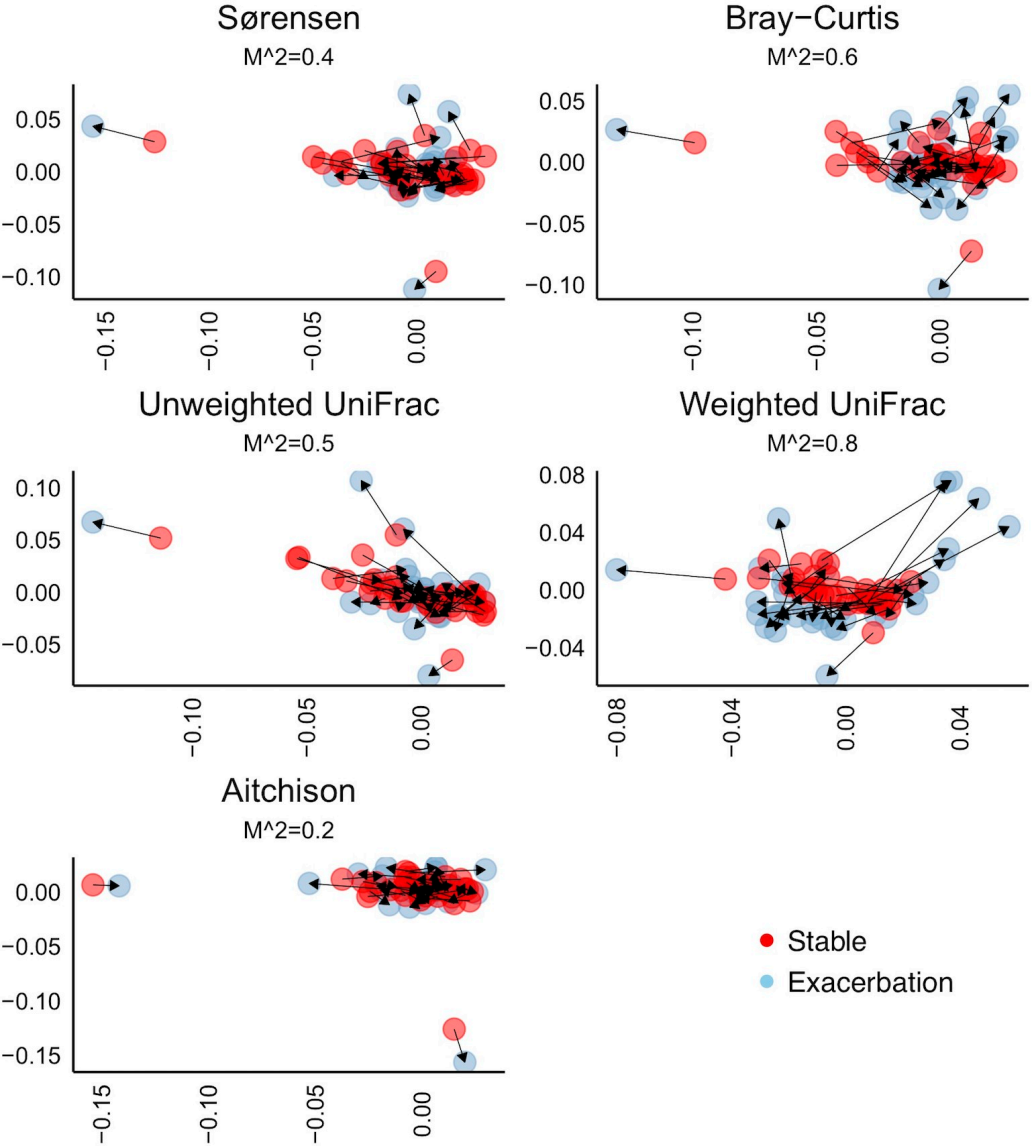
Non-metric multidimensional scaling plots after symmetric Procrustes transformation, illustrating differences in microbiota as distance between paired samples collected at stable state and during exacerbations of COPD. Distance matrices: Sørensen and Bray Curtis: Both non-phylogenetic; qualitative and quantitative information respectively. Unweighted and weighted UniFrac: Both phylogenetic; qualitative and quantitative information respectively. Aitchison: Compositional interpretation of sequencing data. M^2 = Summed squares of distances. The significance of M^2 was tested for all comparisons, with p<0.05 for all but WUF (PROTEST p = 0.25).

To investigate beta-diversity within individuals, one distance matrix was created for each disease state. Overlaying stable state and exacerbation ordinance plots after Procrustes transformation showed ample distance within several sample pairs (Fig 8). M^2 values > 0.3 indicate that the samples delivered at the different disease states have poor resemblance. Information on which pairs have the least similar samples is given in Fig 2 in S1 Text. More on bioinformatics and statistical methods.

### Longitudinal case study

One patient (NN) delivered induced sputum samples from six stable state visits and seven exacerbations. NN was a 66-year old ex-smoker, diagnosed with COPD stage IV at inclusion. NN continued being a frequent exacerbator the three years the study lasted.

**Fig 9:**
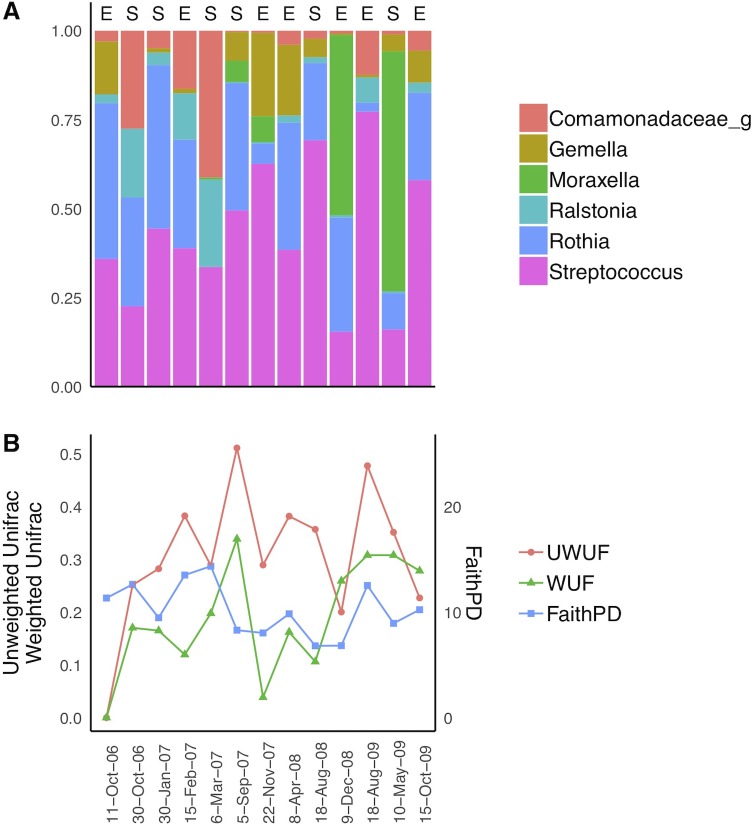
Taxonomic composition and diversity in 13 induced sputum samples collected from the same patient (chronic obstructive pulmonary disease) at different consultations. A: Presenting taxonomic composition at level 6 taxonomy (genus), provided by Silva database for amplicon sequence variants containing at least 1% of all sequences. Numbers are given as relative abundances per sample. B: Phylogenetic alpha- and beta-diversity. Alpha-diversity measured by Faith phylogenetic diversity (right y-axis). Non-quantitative and quantitative beta-diversity measured by UniFrac (UWUF, WUF respectively, left y-axis). Except from the first sampling time point beta diversity is calculated between consecutive samples. A+B: Disease state given in A by S = Stable state, E = Exacerbations. Samples are ordered chronologically and collection dates are given in B.

The taxonomic composition (including ASVs consisting of ≥ 1% of all sequences) for each of the 13 samples are shown in Fig 9A. Of the six dominating genera, *Streptococcus*, *Ralstonia* and *Comamonadaceae* were seen in all samples. *Rothia*, *Moraxella* and *Gemella* were the other genera found to dominate, though not consistently seen at each sampling occasion.

Variability in phylogenetic diversity measures are displayed in Fig 9B. Alpha-diversity changed between sampling time points, but there was no consistent pattern in directionality between the stable state samples and the samples collected during exacerbations. When comparing beta-diversity, there was a trending increase in distances over time with unweighted UniFrac. However, this could not be seen for weighted UniFrac distances, which also varied over time unrelated to disease state.

## Discussion

This study has shown that individual COPD patients had evident changes in the sputum microbiota from stable state to exacerbation, in parallel with significant changes in sputum inflammatory markers. The individual’s changes in microbiota were to some extent camouflaged when analyses were run on groups of patients. Considerable shifts in bacterial composition were seen in the case study over 13 repeated stable state/exacerbation samples, but without a consistent stable state equilibrium.

COPD exacerbations are heterogeneous events, differing in length, symptom burden and need for treatment. In the current study, only patients who met the clinical criteria for an exacerbation, defined by the Wedzicha and Donaldsons’ definition [29] and the judgment of an experienced study physician were included. All patients came to the outpatient clinic by themselves, and only those patients deemed not in need for hospitalization were considered for induced sputum sampling. Thus, all exacerbations were moderate at the time of sampling. Still, the sputum inflammatory markers confirmed an altered local immune state during these events, showing both that the exacerbation state was truly different from the stable state, and also that microbiota likely was affecting, or affected by, the airways inflammation.

We observed significantly higher levels of TNF-α, IP-10 and MIG during exacerbations. TNF-α is an upstream inflammatory cytokine with a wide range of effects. It has an important role in Th1-mediated immune responses, augmenting both IP-10 and MIG signaling downstream [30]. These are cytokines induced by interferon-gamma (IFN-γ) as part of a Th1-mediated immune response [30]. All three cytokines have been shown to play a role against viral infections, intracellular bacteria and to some extracellular bacteria [31–33].

The AMPs are part of the innate immune response against a wide variety of microbes including bacteria, fungi and viruses. In a previous study from the BCCS and BCES, we have shown the same disease state related pattern of change as found in the current study [18]. In patients where the composition of the taxa in sputum changed with disease state, SLPI was significantly lower during exacerbations compared to those patients where the sputum composition was unchanged. Presumably this is a response to the microbial shift, for instance by degradation of SLPI by host and microbial proteases. However, in theory it could also be opposite; that during an exacerbation the immune response leads to changes in taxonomic compositions. In vitro studies are likely necessary to elucidate specific mechanisms. For the other markers, we could not find an association with shifts in taxonomic composition. Low sample size is perhaps the most likely explanation for this, in addition to the inherent heterogeneity of the COPD exacerbations.

The four most abundant phyla in our samples were coherent with previous studies on COPD sputum microbiota [34, 35]. It was the same four phyla dominating the samples independently of disease state, though we did see a shift involving increases in *Proteobacteria* during exacerbations, and a parallel decrease in *Bacteroidetes*. In the previous study by Mayhew et al [34], it was further shown that the fraction of *Proteobacteria* increased with increasing exacerbation severity, something the current study did not have power to examine. However, the current study adds to the other studies by showing an accompanied immune response with the shifts in microbial profiles.

Another important difference between our study and previous studies is that the current study only included induced sputum samples. We have previously shown that induced and spontaneous sputum collected during the same visits will not be sufficiently similar in microbial composition to allow them to be used interchangeably [17].

The most abundant genus belonging to the *Proteobacteria* phylum in our cohort was *Haemophilus*. This was the case for both stable state and exacerbation, and there were no significant changes in its abundance across disease states. Even though several studies have found *Haemophilus* to be of importance related to inflammation and exacerbation risk [36, 37], we could not find that *Haemophilus* discriminated between disease states when measured in induced sputum. This could reflect the sample size in the current study, and should not be interpreted as changes in *Haemophilus* being without importance. An imperative consideration when evaluating taxonomic composition is that increasing levels of one taxon invariably will result in decreases in others, since the sum total is 100%. We have used the Yue-Clayton index (1-θ_YC_) in an attempt to quantify the difference, but the cut-off value of 0.2 is arbitrary and no established consensus on what constitutes a biologically meaningful cut-off value exist. If the entire ecological content of a sample, or the overabundance of one low-abundant pathogen is more relevant to exacerbation risk, then a cut-off of 0.2 may be too high.

With that caveat, a very important finding in the current study was that there appeared to be significant changes in taxonomic composition when we examined individual (paired-samples) changes (1-θ_YC_>0.2, n = 26) again confirming findings by Mayhew et al. However, we did not find significant changes in composition when all samples were pooled by disease state (Aldex2 analyses p>0.05). Thus, paired analyses are necessary to evaluate changes in taxonomic compositions, and they confirm heterogeneity among patients.

With an infectious exacerbation, where the compositional taxonomy changes, one would imagine that the diversity would change as well. If one pathogenic organism dominated, it would presumably displace others completely (leading to a loss in richness) or skew the distribution significantly (leading to a loss of evenness).

In this study, we could not find a significant difference in alpha diversity between disease states on the group level with either non-phylogenetic or phylogenetic indices. However, we did detect higher diversity at stable state in patients with elevated ANC during exacerbations, indicating that reduced diversity can impair systemic immune responses. The plot showing individual changes revealed that alpha-diversity takes on all directionalities with changing disease states, thus larger numbers would be needed to look at sub-types in more detail.

For beta diversity, using several indices we again saw no convincing change in diversity from stable state to exacerbation with group comparisons, while such changes were supported when looking at diversity with paired analyses. Further, the larger change detected with weighted UniFrac than unweighted, could imply that we see predominately a change in pre-existing bacteria rather than addition or loss of new species.

Some methodological shortcomings need to be considered. First, we lack negative controls of the fluids used in the sputum induction in our study. We used the Decontam algorithm in R to identify likely contaminants, which were then excluded from the study. However, the lack of negative controls remains a weakness, as that could possibly have led to a more precise identification of contaminants. Second, over the three years of the study, two different technicians performed the initial processing of the samples, although with the same protocol. And, although the same study personnel later analyzed all samples with the same protocol, all paired samples were not always analyzed on the same laboratory runs. Analyses of taxonomy and beta diversity did not reveal clear differences in relative abundance between runs or significant differences in beta-diversity, and thus no adjustment for runs were used. However, some laboratory induced inter-pair variation cannot be excluded. Third, since the study compares pairs both where the stable state comes prior to the exacerbation and vice versa, the study is a comparison between disease states, and no chronological sequence of events can be assumed. Fourth, the sample size of the study is too small to make inferences about whether some subgroups like patients with different disease severity have larger variations in their microbiota than other subgroups. Fifth, variability between consecutive samples could not be addressed as participants delivered only one sample at each visit. Sixth, sputum was examined and discarded if the number of cells was < 1 million/mL or number of epithelial cells > 20%. However, even if deemed representative of the lower airways, sputum will invariably contain some microbial contamination from the relatively high-biomass oral cavity. For less contamination prone sampling of the lower airways, bronchoscopy is preferred, however, that is not feasible during COPD exacerbations. Seventh, the true stability of the airways’ microbiome is yet unknown, thus some of the change between the stable state and the exacerbations, may just reflect the fluctuating nature of the microbiome. Finally, amplicon sequencing only tells us which bacteria are present and their relative abundance based on the amplicon sequenced (in our case 16S rDNA).

This study not only confirms that there are considerable changes in the respiratory microbiota between disease states in COPD patients, it further shows an important heterogeneity between patient’s microbiota. This is indicative of future challenges in development of applicable anti/pro-biotic treatment for groups of COPD patients. At the same time local inflammation is associated with the changes in microbiota, indicating the microbiota has significant implications for respiratory health. Further mechanistic studies are needed to examine the interaction between the microbiota and local inflammation.

## Supporting information

S1 Text More on bioinformatics and statistical methods. Supplementary details on bioinformatics and statistical methods.
(DOCX)

S1 Table P-values for differential abundances of features between disease states at different taxonomic levels evaluated by use of Aldex2 in R. p*: FDR-corrected p-values, Wilcoxon test. Effect size cut off 0.5.
(XLSX)
