# Supplementary material for: Sputum microbiota and inflammation at stable state and during exacerbations in a cohort of chronic obstructive pulmonary disease (COPD) patients
Source: PLoS One. 2019 Sep 17;14(9):e0222449. doi: 10.1371/journal.pone.0222449 (PMC6748569; doi:10.1371/journal.pone.0222449)
Supplement: S1 Text — Supplementary details on bioinformatics and statistical methods. (DOCX) [file pone.0222449.s001.docx]

# S1 Text. More on bioinformatics and statistical methods.

*Bioinformatics analyses*

The complete 16S rRNA region V3-V4 sequencing results from all 108 induced sputum samples available for sequencing were quality- and chimera filtered through the microbiota pipeline Quantitative Insights Into Microbial Ecology 2 (QIIME2) (v.2017.9 – v.2018.8) [1]. Our samples were processed in our own laboratory at the Department of Clinical Science, University of Bergen; on four different runs on Illumina MiSeq System (Part # 15044223 Rev. B, MiSeq Reagent Kit v3) in 2015. The fastq files were processed in four batches, one per MiSeq run, to avoid mixing different error models as advised with The Divisive Amplicon Denoising Algorithm 2 (DADA2) [2]. Quality scores for forward and reverse reads were visualized separately, and a median Q-score>25 guided the truncating length of each sequence. Primer regions were removed and DADA2 was further used to filter the data (by default settings) and create error models based on the Illumina sequences from each of the four runs. Sequences were organized (de-replicated) in fewer groups of amplicon reads encoding the same sequences. The error models were then used to accurately group sequences into amplicon sequence variants (ASVs). The resulting composition of the samples thus contains information of a central amplicon representing similar amplicons and the total abundances of DNA-strands fulfilling the criteria for similarity. When joining forward and reverse reads the smallest overlap were acceptable with 50 nucleotides. Chimera removal, based on identifying bimeras (sequences originating from the PCR process after combining two parent sequences into a novel sequence), was conducted by combining all samples [2]. Control of data by use of BLAST [3] revealed persistence of chimeric sequences, and VSEARCH was run to further decrease the number of chimera [4]. This reduced the number of ASVs to 2052 (4.681.840 sequences). To assign taxonomy to our ASVs we self-trained a Naïve Bayes classifier by use of the Silva database at the level of 99% sequence similarity [5].

As we did not have negative controls, we used the total DNA-load measurements (Quant-iT™ PicoGreen™, ThermoFisher Scientific Inc) and the R-package Decontam to filter contaminants [6]. Decontam identified 30 contaminating ASVs across 83 of all 108 sputum samples. The contaminants belonged to seven different phyla: *Proteobacteria, Firmicutes, Bacteroidetes, Fusobacteria, Actinobacteria, Saccharibacteria* and *Spirochaetae*. In addition three ASVs not assigned taxonomy were identified as contaminants. 379 ASVs listed either as “unclassified”, or as “Bacteria”, were omitted (9004 of all 4.459.441 sequences). A random control of sequences missing taxonomic assignment against BLAST [3] proved that these were mainly host-derived DNA-sequences.

Finally, we filtered out ASVs containing less than 10 sequences, and ASVs seen in less than five samples. After removal of the small and rare ASVs, the 72 samples included in the study contained 2.8 million sequences and 408 ASVs. The sparsest sample contained 10.401 sequences.

All 108 induced samples were used to build the phylogenetic tree. The central sequence variant (representative sequence) for each ASV was de-novo aligned, filtered to avoid variable positions along the sequences, and FastTree were used to build a rooted tree [7].

*Statistical analyses*

To compare taxonomic compositions between pairs of samples we calculated the Yue-Clayton measure of dissimilarity (1-θ_YC_ - range 0 to 1; 0=perfect similarity, 1=perfect dissimilarity) [8]. This was performed on the dominating taxa (ASVs containing at least 1% of the total amount of sequences) at Silva’s taxonomic level 6; corresponding to genera.

Comparing disease states differential abundances were evaluated in R by use of Aldex2 [9] which allows for comparison of paired samples with n=2 for each participant. We looked for differences at the taxonomic levels of phyla and genera, and for ASVs (see S1Table).

Comparisons of alpha- and beta-diversity were performed on rarefied ASV-tables. To ensure sufficient sequencing depth, rarefaction curves were generated in R (Fig 1 in S1 Text. More on bioinformatics and statistical methods). All samples were included with a rarefaction depth of 10.401 sequences/sample, with little risk of underestimating diversity. The R package Vegan (Adonis) [10] was used to evaluate both alpha- and beta-diversity for the paired design, while QIIME2 was used for the longitudinal evaluation. In Vegan alpha-diversity (within-sample diversity) was evaluated through Faith’s phylogenetic and Shannon’s non-phylogenetic matrices with Wilcoxon signed-rank test. Permutational multivariate analysis of variance (PERMANOVA) was used to compare differences in beta-diversity between stable state and exacerbation when samples were grouped by disease state, both considering the phylogenetic distance matrices weighted UniFrac (WUF) and its qualitative equivalent unweighted UniFrac (UWUF). In addition, we compared the non-phylogenetic distance matrices developed by Bray-Curtis and Sørensen. To consider the compositional nature of sequencing data, we also ran the Aitchison diversity measure [9, 11]. The longitudinal data were evaluated only with phylogenetic matrices (Faith’s phylogenetic diversity, UWUF and WUF). To compare beta-diversity within sputum-pairs, diversity matrices were created for stable state samples and exacerbation samples respectively. Procrustes symmetric rotation and scaling were then used to minimize the distance between paired samples from corresponding matrices and results plotted (Fig 2 in S1 Text. More on bioinformatics and statistical methods). Positioning of the stable state samples relative to other stable state samples are shown, and an arrow gives the distance to the same participants exacerbation state sample after Procrustes transformation. The sum of the squares of these distances (also called residuals or Procrustes M^2) [12], represents the best fit between the matrices, and will be between 0-1. A higher value of M^2 indicates greater differences between the matrices. Symmetric Procrustes analyses were further repeated (999 permutations) using PROTEST in Vegan to estimate significance of the Procrustes statistics [10].

Stata SE (StataCorp. 2014. Stata Statistical Software: Release 14. College Station, TX: StataCorp LP) was used for analyses of clinical data relative to measurements from biological samples. Shapiro-Wilks test were used to evaluate data-distribution and statistical tests chosen accordingly, also with consideration of paired data. For evaluating white blood cell counts and absolute neutrophil counts against alpha-diversity in sputum the serum markers were categorized in Stata as high or low with cut offs at 11.3 10^9^/L and 8.4 10^9^/L.

## Supplementary table and figures

| Table 1. Alpha-diversity in induced sputum collected  at stable state and exacerbations in the same COPD patients. | | | | |
| --- | --- | --- | --- | --- |
|  | Stable state | Exacerbation | p* |  |
| Faith´s PD |  |  |  |  |
| median (IQR) | 13.8 (12.1-14.9) | 13.6 (12.4-14.5) | 0.7 |  |
| Shannon |  |  |  |  |
| median (IQR) | 4.5 (3.8-4.8) | 4.3 (3.9-4.7) | 1 |  |
| *Wilcoxon signed rank test. COPD: Chronic obstructive pulmonary disease. PD: Phylogenetic diversity. IQR: Interquartile range | | | |  |

Fig 1: Rarefaction curves for 72 sputum samples collected from patients suffering from COPD at stable state and during exacerbations.

Fig 2. Beta-diversity in induced sputum sampled from 36 COPD patients at different disease-states, compared using Procrustes transformation of stable state and exacerbation matrices. Each participant’s sputum pair is listed on the x-axis. For each pair of sputum the Procrustean residuals are calculated (the squared distance between the samples forming a pair, also called Procrustes M^2). Distance matrices: Sørensen and Bray Curtis: Both non-phylogenetic; qualitative and quantitative information respectively. Unweighted and weighted UniFrac: Both phylogenetic; qualitative and quantitative information respectively. Aitchison: Compositional interpretation of sequence counts. The horizontal line marks the median. Dotted lines mark the interquartile range (IQR).

## References

1. Bolyen E RJ, Dillon MR, Bokulich NA, Abnet C, Al-Ghalith GA, Alexander H, et al. QIIME 2: Reproducible, interactive, scalable, and extensible microbiome data science. PeerJ Preprints. 2018;6:e27295v2. doi: 10.7287.

2. Callahan BJ, McMurdie PJ, Rosen MJ, Han AW, Johnson AJ, Holmes SP. DADA2: High-resolution sample inference from Illumina amplicon data. Nat Methods. 2016;13(7):581-3. doi: 10.1038/nmeth.3869. PubMed PMID: 27214047; PubMed Central PMCID: PMCPMC4927377.

3. Agarwala RBT, Beck J, Benson DA, Bollin C, Bolton E, Bourexis D, et al. Database resources of the National Center for Biotechnology Information. Nucleic Acids Res. 2018;46(D1):D8-D13. doi: 10.1093/nar/gkx1095. PubMed PMID: 29140470; PubMed Central PMCID: PMCPMC5753372.

4. Rognes T, Flouri T, Nichols B, Quince C, Mahe F. VSEARCH: a versatile open source tool for metagenomics. PeerJ. 2016;4:e2584. doi: 10.7717/peerj.2584. PubMed PMID: 27781170; PubMed Central PMCID: PMCPMC5075697.

5. Quast C, Pruesse E, Yilmaz P, Gerken J, Schweer T, Yarza P, et al. The SILVA ribosomal RNA gene database project: improved data processing and web-based tools. Nucleic Acids Res. 2013;41(Database issue):D590-6. doi: 10.1093/nar/gks1219. PubMed PMID: 23193283; PubMed Central PMCID: PMCPMC3531112.

6. Davis NM, Proctor DM, Holmes SP, Relman DA, Callahan BJ. Simple statistical identification and removal of contaminant sequences in marker-gene and metagenomics data. Microbiome. 2018;6(1):226. doi: 10.1186/s40168-018-0605-2. PubMed PMID: 30558668; PubMed Central PMCID: PMCPMC6298009.

7. Price MN, Dehal PS, Arkin AP. FastTree: computing large minimum evolution trees with profiles instead of a distance matrix. Mol Biol Evol. 2009;26(7):1641-50. doi: 10.1093/molbev/msp077. PubMed PMID: 19377059; PubMed Central PMCID: PMCPMC2693737.

8. Yue JC, Clayton MK. A similarity measure based on species proportions. Commun Stat Theory Methods. 2005;34(11):2123-31. doi: 10.1080/sta-200066418.

9. Gloor GB, Reid G. Compositional analysis: a valid approach to analyze microbiome high-throughput sequencing data. Can J Microbiol. 2016;62(8):692-703. doi: 10.1139/cjm-2015-0821. PubMed PMID: 27314511.

10. Oksanen J, Friendly M, Kindt R, Legendre P, McGlinn D, Minchin PR, et al. vegan: Community Ecology Package. R package version 2.5-2. 2018.

11. Gloor GB, Macklaim JM, Pawlowsky-Glahn V, Egozcue JJ. Microbiome Datasets Are Compositional: And This Is Not Optional. Front Microbiol. 2017;8:2224. doi: 10.3389/fmicb.2017.02224. PubMed PMID: 29187837; PubMed Central PMCID: PMCPMC5695134.

12. Gower JC. Generalized procrustes analysis. Psychometrika. 1975;40(1):33-51.
